# 3D Printing and 3D Bioprinting in Pediatrics

**DOI:** 10.3390/bioengineering4030063

**Published:** 2017-07-13

**Authors:** Sanjairaj Vijayavenkataraman, Jerry Y H Fuh, Wen Feng Lu

**Affiliations:** Department of Mechanical Engineering, National University of Singapore (NUS), Block EA 02-17, 9 Engineering Drive 1, Singapore 117576, Singapore; mpefuhyh@nus.edu.sg (J.Y.H.F.); mpelwf@nus.edu.sg (W.F.L.)

**Keywords:** additive manufacturing, 3D printing, bioprinting, pediatrics

## Abstract

Additive manufacturing, commonly referred to as 3D printing, is a technology that builds three-dimensional structures and components layer by layer. Bioprinting is the use of 3D printing technology to fabricate tissue constructs for regenerative medicine from cell-laden bio-inks. 3D printing and bioprinting have huge potential in revolutionizing the field of tissue engineering and regenerative medicine. This paper reviews the application of 3D printing and bioprinting in the field of pediatrics.

## 1. Introduction

3D printing or additive manufacturing (AM) is a process of fabricating three dimensional solid objects from a 3D model or digital file. Additive manufacturing consists of several techniques to build 3D objects layer by layer, which are grouped under seven categories by American Society for Testing and Materials (ASTM) Committee F42 on Additive Manufacturing Technologies, as shown in Table 1 [1]. The description of each process is also given in the table.

**Figure 1:**
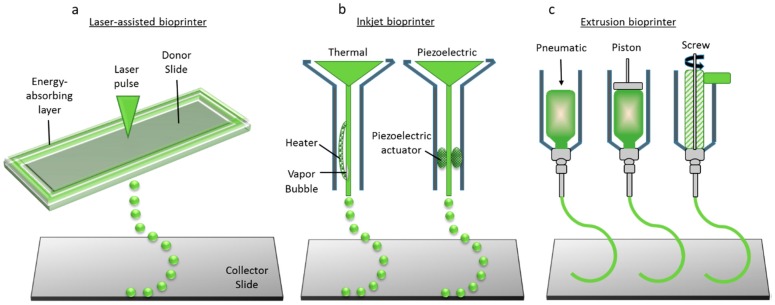
Three main bioprinting technologies: (**a**) Laser-assisted bioprinting; (**b**) Inkjet printing; (**c**) Extrusion or robotic dispensing bioprinters (adapted from Reference [3]).

Bioprinting is defined as the use of 3D printing technology with materials that incorporate viable living cells, e.g., to produce tissue for reconstructive surgery [2]. Biopolymers or cell-laden hydrogels are arranged spatially in a 3D dimensional pattern and built layer by layer into a tissue or organ. The three main bioprinting techniques are laser-assisted bioprinting, inkjet bioprinting, and extrusion bioprinting [3,4], as shown in Figure 1. Laser-assisted bioprinting focuses laser pulses on to the donor slide, thus creating high pressure to propel droplets of cell-laden hydrogel on to the collector slide. Inkjet printing ejects droplets of biopolymer or cell-laden hydrogels through a nozzle by either thermal energy application (electrically heating to produce vapor bubbles that forces droplets to come out through the nozzle) or a piezoelectric actuator (actuation of piezoelectric crystals by applying electrical energy at high frequencies). Extrusion or robotic dispensing bioprinters extrude biopolymers or cell-laden hydrogels through the nozzle by applying air pressure (pneumatic) or mechanical systems (piston or screw). The pros and cons of these three types of bioprinting processes are given in Table 2. Though bioprinting is a potential technology for tissue engineering and regenerative medicine, there are many ethical, legal, and social concerns which are to be overcome before it can be successfully put into clinical use [4,5].

Material selection is key for the successful application of AM and bioprinting techniques. The choice of material depends on the intended application. For the fabrication of 3D organ models for surgical planning, the resolution of the to-be printed model determines the AM technique to be used and hence, the material. If one of the Vat Polymerization processes such as SLA were used, then the material would be a photopolymer. Here, the resolution required determines the process and the materials, as their intended use is for surgery planning and training. However, the selection of materials becomes a critical step when it comes to tissue or organ printing. For the bioprinting of soft tissues such as skin, natural polymer-based hydrogels such as collagen, gelatin, and chitosan are used. On the other hand, for hard tissues such as bone, materials with better mechanical properties are preferred to meet the functional tissue requirement. Hence, synthetic polymers such as Polycaprolactone (PCL) and naturally occurring minerals such as hydroxyapatite (HA) are used for bone tissue engineering [6,7,8].

## 2. Applications in Pediatrics

Applications of AM and 3D bioprinting in the field of pediatrics are diversified. The three main application categories are: (i) Surgical planning, (ii) Prostheses, (iii) Tissue constructs, and (iv) Drug printing. The applications of AM and bioprinting in these three categories are discussed shortly in the sections below.

### 2.1. Surgical Planning

**Figure 2:**
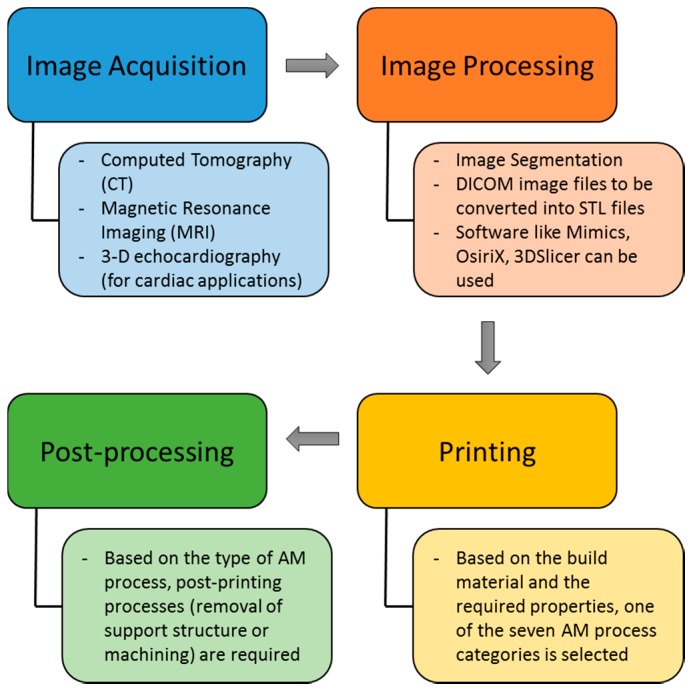
Steps involved in the fabrication of 3D models using Additive Manufacturing (AM) technologies.

Surgical planning is the pre-visualization of a surgical intervention using virtual or visual aids such as Computed Tomography (CT)/Magnetic Resonance Imaging (MRI) images, and 3D models in order to ensure that the surgical steps are well planned and predefined so as to aid in a smooth surgery. Neurosurgery as well as oral and maxillofacial surgery require extensive pre-planning, making surgical planning a critical pre-operative procedure. Surgical planning becomes critical when it comes to pediatric patients. The steps involved in fabricating a 3D model for surgical planning using AM technology is given in Figure 2. These steps are also common in prosthetics fabrication.

The first step is image acquisition. Computed tomography (CT) and Magnetic Resonance Imaging (MRI) are the two most widely used imaging modalities. 3D echocardiography has also been recently explored. The acquired images cannot be directly used and require processing before they can be sent to the 3D printer. The first step in image processing is the segmentation process, where the blood pool is segmented from the organ anatomy. Though software such as Mimics and OsiriX are helpful in segmentation, extensive manual work using drawing, erasing, and regional thresholding tools in addition to interpolation of the data between the slices are required, especially when the boundary between the blood pool and the myocardium is not readily recognizable [9]. The DICOM (Digital Imaging and Communication in Medicine) files are then converted into STL (Stereolithography or Standard Tessellation Language) file format. The STL file can then be sent to the 3D Printer to fabricate the physical 3D model. The type of 3D printing process to be selected is based on the material and required properties from one of the seven AM categories. After the 3D model is fabricated, based on the AM process, some post-processing is required to obtain the final 3D anatomical model. Post-processing might include the removal of a support structure (for parts that have overhangs or for those processed by AM methods that require support structure such as FDM), removal of powder sediments with waterjet techniques (for powder-based AM processes), and finishing processes, if applicable and required, including bead-blasting, tumble-finishing, plating, and painting for enhancing the surface characteristics and aesthetics [10]. While additively manufactured polymer parts could be used as-printed, metal and ceramic parts require post-processing to achieve acceptable surface finish, form accuracy, and material properties [11].

#### 2.1.1. Congenital Heart Disease (CHD)

Most of the applications of 3D printing in pediatric surgical planning reported in the literature is for the surgical planning of CHD [12]. Children have much smaller hearts than adults due to their smaller chest cavities and, together with the complexity of certain congenital heart defects, this makes congenital heart surgery more challenging compared with adult heart surgery. Additive manufacturing comes in handy for the pre-visualization and pre-planning of the surgical procedures for complex pediatric heart surgeries. There are many successful cases demonstrating the potential of additive manufacturing in pediatric surgical planning. CT angiographic data were used to design the 3D heart models of patients with pulmonary atresia (with ventricular septal defect) and major aorto-pulmonary collateral arteries; these models were used by the surgeons for preoperative and intraoperative planning [13]. The surgeons found the models to be very useful in visualizing the vascular anatomy, and the 3D models accurately represented the major aorto-pulmonary collateral arteries identified during surgery and conventional angiography, by 96% and 93%, respectively. Rigid and flexible pediatric heart models are fabricated by Noecker et al. [14] using stereolithography and 3D printing, to aid in understanding the complex structure and provide a tactile representation of the complex anatomy. In another study [15], the stereolithography method was used to fabricate a 3D heart model of a 3-month-old patient with a sub-pulmonary ventricular septal defect showing the exact dimensions of the defect for surgical planning. One of the most technically challenging surgical procedures is the stenting of a hypoplastic transverse arch, where the risk of post-surgical complications such as stent migration and partial obstruction of the origin of the head and neck vessels are high. A 3D-printed anatomical model was used in the pre-planning of such a complicated surgery of a 15-year-old boy with hypoplastic aortic arch, in order to assess the optimal stent position, size, and length, and was reported to be highly helpful in planning endovascular stenting [16]. Cardiac surgery in patients who have already undergone several reoperations are highly risky. One specific example is the heart transplantation procedure in patients with failing staged palliation after Norwood stage I operation, a Glenn superior cavopulmonary anastomosis, or a Fontan completion operation. In such cases, the surgical planning is extremely complicated and becomes critical. CT and MRI data were used to construct 3D digital models, and anatomical models were fabricated using stereolithography to plan the heart transplantation surgical procedure of two patients (a 2-year-old boy with failing staged palliation of hypoplastic left heart syndrome and a 14-year-old girl who had pulmonary atresia and a hypoplastic right ventricle) [17]. These physical models allowed the surgeon and the pediatric cardiologist to develop the optimal surgical approach during heart transplantation and to anticipate problems that may arise during the dissection or implantation of the heart. The specific dimensions and distances can be measured, and heart transplantation can be planned preoperatively.

#### 2.1.2. Other Applications

AM technology is also applied successfully in the planning of brain surgeries by neurosurgeons. The surgical/endovascular team at the Boston Children’s Hospital used 3D printing to fabricate multiple models for each pediatric patient with arteriovenous malformations (AVMs), with each construct designed to illustrate different aspects of the specific lesion using MRI and CT data. Intraoperative validation of model fidelity was performed using perioperative imaging, surgical filming, and post hoc analysis of models with intraoperative photography [18]. Anatomical models helped in resecting the AVMs without any complication and resulted in a 30-min reduction in operative time (12%) in two cases when they were compared with matched controls. A 3D-printed tracheobronchial tree model fabricated from the CT data of a 1-year-old girl was successfully deployed for the first time in training the clinicians in pediatric bronchoscopy [19]. This is a significant step in bronchoscopy, because the tracheobronchial tree models used for training to date were scaled for adult lungs, and there is a significant variation between adult lungs and the lungs of a neonate or an infant. Besides improving the accuracy, speed, and safety of pediatric bronchoscopic procedures, 3D-printed pediatric anatomical models can also be used to study rare airway pathologies in children or interventional procedures. Another interesting AM application reported is the prenatal evaluation of complex patient-specific fetal anatomy that was subsequently used to manage complex perinatal airway anomalies [20]. The craniofacial anatomy of a fetus was 3D-printed using fetal MRI and computer-aided modelling. Prenatal ultrasound of the fetus indicated a potential upper airway obstruction from a midline mass of the maxilla, while the 3D-printed model indicated the oral airway to be patent, with the mass being isolated in the upper lip and maxilla. The planned ex utero intrapartum treatment procedure was aborted based on the 3D model and the neonate was born with a protuberant cleft lip and palate deformity, without airway obstruction, as predicted by the patient-specific model. Anatomical model, in this case, prevented the surgeons from doing any unnecessary surgical procedure, and the child was discharged without need for airway intervention. Jones et al. [21] developed and validated a physical model to investigate the biomechanics of infant head impact, which is the single most common cause of death or permanent disability from injury in children. Pediatric head injury cause and effect is poorly understood, as the only source of data for such studies have been infant postmortem human surrogates (PMHS). Images acquired from postmortem computer tomography (PMCT) imaging were processed using Mimics Software and a multi-material physical model was 3D-printed using Polyjet 3D printing technology. Significant similarities in responses were reported on the validation of the 3D-printed head model with PMHS data, suggesting a better way to characterize and understand infant head impact injury mechanics that will aid in better clinical management and injury prevention strategies. 

In addition to surgical planning, 3D printing has also been used to make navigation templates that aid surgeons to guide the surgical insertion of internal fixation screws and plates during surgery. A 3D-printed navigation template was used in the surgical procedure for older children with developmental dysplasia of the hip (DDH) [22]. The use of 3D-printed navigation templates resulted in reduced operation time, decreased intraoperative X-ray exposure and surgical risk, reduced epiphysis damage, as well as better operative guidance and surgical precision. Another study used a 3D-printed drill template, made of medical-grade polylactic acid (PLA), for the placement of screws in Locking Compression Pediatric Hip Plate (LCP-PHP) [23], and reported reduced intraoperative damage to the femoral neck epiphysis, decreased operation time, reduced intraoperative hemorrhage, and decreased radiation exposure to patients and personnel during the surgery.

#### 2.1.3. Strengths and Limitations of 3D-Printed Organ Models

Additive manufacturing techniques (listed in Table 1) are used for the fabrication of organ models. Depending on the requirements (complexity of the model, resolution, material, etc.), different AM techniques are adopted. With the development of AM technology, it is now possible to fabricate multi-material, multi-color 3D objects using a multi-nozzle AM system. These developments might be more useful for clinicians to differentiate different anatomical features of the organ model. For instance, the nerves, the blood vessels, the bone can be rendered in different colors. A recent study to evaluate the effectiveness of 3D models on the learning or training process of pediatric residents [24] concluded that the learners’ satisfaction was improved with 3D models compared to that of 2D drawings, around the topic of congenital heart disease, specifically with tetralogy of Fallot. In another study [25], a 3D-printed model of pygopagus conjoined twin anatomy significantly enhanced surgeon understanding of the scale, shape, and correct identification of difficult anatomical structures compared to CT data. Also, the time consumed for such a better anatomical understanding from 3D models was significantly less than that required when using traditional images. However, there are certain limitations. The first limitation is the long processing time and high cost [26,27]. While the material costs for 3D printing are cheap, the 3D printer itself might be expensive and, if the product development costs including design, assembly, testing, and fitting the prostheses are considered, the overall cost will be significantly higher than just the material cost [27]. Secondly, the 3D models now fabricated are rigid or static models that do not allow for the reproduction of physiologic changes occurring during the cardiac cycles; hence, dynamic models are required [12]. The lack of standardization and limitations in the imaging systems are other limitations to note. Moreover, the risk of radiation exposure and the need for sedation during the imaging process also has to be taken into account. Radiation-free imaging techniques and ultra-fast image data acquisition systems that eliminate the need for sedation will facilitate the use of AM technologies in pediatric surgery planning.

### 2.2. Prostheses

A prosthesis is a device that is designed to replace a missing part of the body or to enhance the functionality of any part of the body. Common prosthetic devices include arms, hands, legs, joints, and even diseased eyes. Dental prostheses include false teeth and maxillofacial prostheses include the artificial replacement of the jaw bone. AM technologies are increasingly being used for the fabrication of all the different kinds of prostheses stated above. The advantages of 3D-printed prostheses over conventional ones are in terms of customizability and cost. Furthermore, 3D-printed prosthetics address the unique challenges posed by pediatric prosthetic needs. Due to rapid physical growth, pediatric prostheses become outsized frequently. Also, due to psychosocial development, there are changing needs. Technological advances increase the complexity and weight of the prostheses and hence incur a higher cost. AM can be used to fabricate rugged, light-weight, easily replaceable, and very low-cost prostheses for children [28]. 

#### 2.2.1. Hand Prostheses

One perfect example of 3D-printed prostheses in pediatrics is the prosthetic arm or prosthetic limb. 3D modelling and AM techniques were used to develop an electronic prosthetic hand [29]. The use of open source software and hardware helped in keeping the cost of these electronic prosthetic arms competitive. The electronic prosthetic hand was digitally designed to reconstruct a left artificial hand. Zuniga et al. [30] studied the anthropometric, active range of motion (ROM), and strength changes after six months of using a wrist-driven 3D-printed transitional prosthetic hand (Cyborg Beast transitional prosthetic hand) for children with upper-limb deficiencies. Five children (two girls and three boys, 3–10 years of age) with absent digits (one traumatic and four congenital) participated in this study and were each fitted with a 3D-printed transitional hand prosthesis. Results indicated that there was significant improvement in ROM by using the Cyborg Beast prosthetic hand. Hofmann et al. [31] emphasized the importance of modularity in prosthetic design and argued that such a modular approach needs to consider not only the socket and end-effector but also the extensions that capture significant parameters (such as length, angle, and rotation). A few organizations such as e-NABLE (http://enablingthefuture.org), Open Bionics (https://www.openbionics.com), and NotImpossible labs (http://www.notimpossible.com) provide low-cost arm and hand prostheses for children at a much lesser price than the standard titanium artificial prostheses. 

#### 2.2.2. Other Prostheses

3D-printed leg prostheses are successfully being fabricated by several companies including bionic leg prostheses by BionX Medical Technologies, Inc., Bedford, MA, USA (http://www.bionxmed.com), Exo-Prosthetic leg, San Francisco, CA, USA (https://www.behance.net/gallery/20696469/Exo-Prosthetic-Leg), and Andiamo leg prostheses, London, UK (http://andiamo.io). 3D-printed prosthetic eyes are being developed by the British company Fripp Design and Research, London, UK (http://www.frippdesign.co.uk), using a Z-Corp 510 machine (a powder-based AM technique, 3D Systems Inc., Valencia, CA, USA), at a production rate much faster than existing handmade versions and at a cost reduced by 97%. The main advantages of the 3D-printed prosthetic eyes are the reduced time (from 4–8 h per eye to 150 eyes per hour), reduced cost (£3000 to £100), biomimetic structure (with intricate colored details including the iris and blood vessels) and reproducibility (no variation in quality). A 3D face prosthesis is also another application, where a patient with a part of the face removed due to cancer was fitted with a partly 3D-printed prosthesis serve as a case study [32]. Furthermore, a 3D-printed transparent facemask, fabricated with OBJET MED610 (Stratasys Ltd., Eden Prairie, MN, USA), and lined with two layers of transparent medical silicone gel, was recently used for the treatment of pediatric facial hypertrophic scars after burns [33]. The results indicated that the 3D-printed facemask is an effective treatment method with a decrease in average scar thickness in two patients and a reduction the number of clinical procedures, easing the production and reducing the time consumption of these processes. 

#### 2.2.3. Strengths and Limitations of 3D-Printed Prostheses

3D-printed prosthetics are a boon to pediatric patients because they quickly outgrow prostheses, and the low cost of 3D printing makes repairs and upgrades affordable [34,35]. The design and color of the prosthesis could be chosen to the liking of the pediatric patient to have a positive psychosocial influence. However, care should be taken to ensure that children receive proper fitting, training, and follow-up with a multidisciplinary team to ensure success. Although Davids et al. [36] documented the benefits of fitting children with upper extremity prostheses before the age of 3 years, many 3D-printed devices are not recommended for children under 4 years old because of their often-limited ability to express discomfort and the fact that free distribution of these devices is often not monitored by a health-care professional [34]. The resolution of the 3D printer might be limited to be able to fabricate prostheses for very young children, with smaller parts and hardware. Durability, environment, and lack of printing standards for the manufacturing of 3D-printed prostheses are other factors to consider [30]. In addition to the unique challenges associated with pediatric prostheses, there are other limitations with prostheses in general. Though there is a commendable progress in the fabrication of biomimetic prostheses, in terms of their structure and function, the current prostheses lack the ability to communicate with the brain; they cannot be controlled by the brain signals nor relay sensory data back to the brain. With the advent of bioprinting, cellular prostheses could be an interesting area of research, which would help prostheses to be integrated in the brain communication system, and move their position from that of a prosthesis to exhibit more biomimicry with tissue and organ functionalities. 

### 2.3. Tissue Constructs

3D bioprinting can also be used to fabricate tissue constructs for regenerative medicine in pediatrics. Of the three bioprinting processes (shown in Figure 1), extrusion-based bioprinters are the most common. Many different tissues have been successfully bioprinted as reported in many journal articles [3,37,38], including bone, cartilage, skin, and even heart valves. However, it is important to note that while the published literature on bioprinted tissues and organs are at the laboratory level, there is a long way to go to achieve successful clinical translation [4,5]. There are many detailed reviews published on this subject [39,40,41,42]. Since there are detailed reviews of bioprinting tissues and organs already published and there are no papers pertaining specifically to pediatric tissue printing, the same is not reviewed here. However, bioprinting is a potential technology that has wide applications in pediatrics as well. There are many associated challenges to be overcome before bioprinting could be used for fabricating living pediatric tissue and organs. In addition to the challenges faced by bioprinting in general, such as vascularization and innervation, scalability and quality assurance, the greatest challenge in its application to pediatrics is that the tissue construct or the organ that is fabricated by bioprinting should be able to grow along with the child. Inability to grow will necessitate replacing the tissue construct or organ periodically, involving huge risks and complications. Recent proof-of-principle studies on the 3D printing of self-expandable and biodegradable polymer stents with growth potential (the ability to grow with the patient) [43] demonstrate that earnest efforts are being taken to overcome this challenge. Bioprinting fully functional tissues and organs, with all the biological functions mimicking the native tissue is another challenge. There are also other ethical and legal hurdles to be overcome for the successful clinical translation of this technology [4,5]. 

### 2.4. Drug Printing 

The U.S. Food and Drug Administration (FDA) approved Aprecia Pharmaceuticals Company’s 3D-printed SPRITAM levetiracetam for oral use in treating epileptic seizures recently (August 2015), which furthers the prospect of tailor-made drugs that are customized to individual patient needs [44]. The vision behind AM is that medication will be customized to individuals in ways that make it safer and more effective. Norman et al. [45] delineates three unique attributes where 3D printing distinguishes itself from traditional manufacturing processes: product complexity, personalization, and on-demand manufacturing. The relevance of drug printing to pediatrics in terms of these three unique attributes of AM are discussed below.

#### 2.4.1. Product Complexity

Product complexity refers to the geometrical flexibility with 3D printing. Geometrical flexibility includes size, dose, appearance, and the rate of delivery of a drug based on patient-specific needs. Especially for children, drug printing is a boon. To increase the compliance rate and reduce the resistance of taking medication in children, 3D printing offers a choice for children to choose the color, shape, and design of a tablet. A team of researchers from the University College London School of Pharmacy has suggested that 3D printing can be used to fabricate tablets in any shape (such as animals), and so could potentially increase compliance for pediatric patients [46]. Since the printer software allows the creation of shapes with equivalent volume, tablets of different shape but containing the same dose, can be printed. Printing tablets in different shapes and colors to the liking of the child is expected to increase compliance for pediatric patients. It is also important to note that the shape of the tablet affects many other attributes including the disintegration and rate of dissolution or rate of drug release. For instance, SPRITAM^®^, an FDA-approved 3D-printed drug, has a unitary porous structure produced by a 3D printing process that binds powders without compression. This structure allows tablets with up to 1000 mg of levetiracetam to disintegrate within seconds when taken with a sip of water [44].

#### 2.4.2. Personalization

Based on a patient’s mass and metabolism, the amount of drug delivered has to be tailored, which is called personalization. 3D-printed dosage forms could ensure accurate dosing in growing children and permit personalized dosing of highly potent drugs such as theophylline and prednisolone [45]. Also, the concept of ‘polypills’, which is a single pill consisting of the entire patient’s medication, can be realized with AM. An inexpensive desktop 3D printer was used to fabricate relatively complex formulations into bilayer tablets that match the release of a commercial guaifenesin bi-layer tablets (GBT) (manufactured using conventional tablet compression methods) [47]. This is especially beneficial to children in improving the compliance to medication as one pill replaces multiple pills.

#### 2.4.3. On-Demand Manufacturing

Drugs can be printed on-demand at the point of care using AM. In time-constrained and resource-constrained settings such as natural disasters, military operations, emergency and operating rooms, and critical care units, on-demand drug printing will be of immense use [45]. For children, it means they could choose the shape and color of the tablet for every dose, especially pediatric patients with chronic illness. Additionally, for low-stability drugs, AM is very beneficial. For instance, a drug named 1,2,3-trinitroxypropane (nitroglycerin) that is used in the treatment of angina pectoris has a tendency to degrade upon storage [48]. Such low-stability drugs, if manufactured on-demand, could reduce this issue significantly.

## 3. Conclusions

Additive manufacturing and 3D bioprinting have many potential applications in the field of pediatrics. They possess numerous advantages and offer unique possibilities compared to the existing technologies. The three main application areas of AM and bioprinting in pediatrics are surgical planning, prosthetics and tissue constructs, and drug printing. While various AM techniques are used to fabricate surgical models for the pre-planning of surgical procedures, the fabrication of customized patient-specific prostheses, and the printing of drugs/tablets, bioprinting is used to fabricate cell-laden tissue constructs. While the technology growth is commendable, the associated ethical and legal challenges are not addressed in commensurate, which will delay the clinical translation. There is a huge potential to utilize AM and bioprinting in pediatrics, yet to be explored.

## Figures and Tables

**Table 1:** Categories of Additive Manufacturing Technologies.

Category	Description	Examples
Vat Polymerization	Liquid photopolymer in a vat is selectively cured by light-activated polymerization	Stereolithography (SLA), micro-SLA, Digital Light Processing (DLP)
Material Jetting	Droplets of build material are selectively deposited	Objet PolyJet, 3D Systems Projet
Binder Jetting	Liquid bonding agent is selectively deposited to join powder materials	Zcorp, Voxeljet, ProMetal/ExOne
Material Extrusion	Material is selectively dispensed through a nozzle or orifice	Stratasys Fused Deposition Modeling (FDM)
Powder Bed Fusion	Thermal energy selectively fuses regions of a powder bed	Selective Laser Sintering (SLS), Selective Laser Melting (SLM)
Sheet Lamination	Sheets of material are bonded to form an object	Laminated Object Manufacturing (LOM)
Directed Energy Deposition	Focused thermal energy is used to fuse materials by melting as they are being deposited	Laser Engineered Net Shaping (LENS)

**Table 2:** Categories of Bioprinting Technologies.

Category	Materials	Pros	Cons
**Laser-assisted bioprinting**	Cells in media	High accuracy	Low structural integrity
		High resolution	Long printing time
		Capable of single-cell level control printing	Low scalability
**Inkjet printing**	Liquids, Hydrogels	High throughput (Scalable)	Low structural integrity
		High cell viability	Moderate accuracy
		Affordable	Moderate precision
**Extrusion or robotic dispensing bioprinting**	Hydrogels, Cell aggregates	High structural integrity	Low accuracy
		Short printing time	Low precision
		Multi-nozzle multi-material printing feasible	Cells undergo shear stress at nozzle tip
